# Prolonged exertion of self-control causes increased sleep-like frontal brain activity and changes in aggressivity and punishment

**DOI:** 10.1073/pnas.2404213121

**Published:** 2024-11-11

**Authors:** Erica Ordali, Pablo Marcos-Prieto, Giulia Avvenuti, Emiliano Ricciardi, Leonardo Boncinelli, Pietro Pietrini, Giulio Bernardi, Ennio Bilancini

**Affiliations:** ^a^Molecular Mind Laboratory, Institutions, Markets, Technologies School for Advanced Studies Lucca, 55100 Lucca, Italy; ^b^Laboratory for the Analysis of compleX Economic Systems, Institutions, Markets, Technologies School for Advanced Studies Lucca, 55100 Lucca, Italy; ^c^Department of Economics and Management, University of Florence, 50127 Firenze, Italy

**Keywords:** ego depletion, mental fatigue, local sleep, punishment, aggressive behavior

## Abstract

Impulsive reactions in social interactions may result in poor or even detrimental outcomes. Particular cognitive states, such as mental fatigue induced by extended practice with cognitively demanding activities, especially if combined with sleep restriction or deprivation, seem to impair the individuals’ ability to exert self-control effectively and may result in impulsive behaviors, including aggressive acts. We demonstrate that exertion of self-control for as little as 45 min can lead to an increased propensity for engaging in aggressive acts in the context of socially relevant choices, as measured by a set of economic games. Also, we show that such behavioral changes are associated with increased sleep-like (delta) activity within frontal brain areas related to decision-making and impulse control. The local occurrence of sleep-like slow waves may lead to a disengagement of frontal areas and explain the reduced ability of individuals to exert self-control effectively. Our results suggest that sleep-like activity may emerge within the awake brain within a relatively short time scale, with detectable effects on socially relevant behavior.

## Significance

In this paper, we demonstrate that prolonged exertion of self-control via cognitively demanding tasks induces a state of fatigue marked by the emergence of sleep-like brain activity within the prefrontal cortex. While they were in this state, individuals displayed an increased propensity to behave aggressively during economic games that simulated socially relevant scenarios. Specifically, we observed a heightened hostility in the Hawk and Dove game and a marked tendency for spiteful punishment in the Public Goods Game. These findings indicate that the propensity for prosocial behavior may be reduced in states of cognitive fatigue resulting from the extended exertion of self-control.

When making decisions in everyday life, we are inevitably shaped by contingent physiological, psychological, and cognitive factors. As a result, sometimes we make decisions that may appear inconsistent with our usual social behavior, even leading to unexpected aggressive behavior instead of a more desirable cooperative one (1, 2). Among the cognitive factors underpinning social behavior, the tension between intuition and deliberation plays a major role in determining cooperative or antisocial choices. Studies that investigated the role of this factor typically did so by combining cognitive manipulations and economic games (EG) (3–7). Many of such studies advocated a positive effect of intuition on cooperation (8–10), while others supported a central role of deliberation in cooperative behavior (11, 12), with antisocial acts (including criminal acts) assumed to have a more impulsive nature instead (13).

Among the cognitive manipulations used to affect the balance between intuition and deliberation, ego depletion is one of the most popular and controversial (14). In a typical ego depletion paradigm, cognitively demanding tasks requiring participants to use self-control and decision-making (so-called “executive”) functions are employed under the assumption that such an effort will deplete willpower resources (14–16). Tasks adopted in the literature include for instance, Stroop tasks, emotion suppression, and other various high-conflict decision-making tasks (14, 17, 18). After such manipulations, individuals asked to complete a subsequent task requiring the same cognitive resources showed impaired executive functions and greater reliance on a more automatic and intuitive course of action (19–22).

However, results concerning the ego depletion effect have been strongly criticized due to inconsistent replication across studies (23). Indeed, while some investigations, including a large multisite replication study, failed to obtain solid evidence in support of an ego depletion effect (24), a reanalysis of the same data and other works indicated that some behavioral manipulations may be more efficient than others at inducing ego depletion (25, 26). Moreover, some authors noted that the literature includes little or no evidence in net contrast with the ego depletion hypothesis [i.e., reporting that manipulations improved self-control; (23)]. Overall, these observations may be taken to indicate ego depletion as lying along a spectrum in which the strength of the effect depends on the efficacy and/or duration of the applied manipulation.

Extended task practice leads to the appearance of slow electroencephalographic (EEG) waves in task-related areas (27–29). These waves occur in the delta (1 to 4 Hz) and/or theta (4 to 8 Hz) frequency ranges and closely resemble the slow waves that characterize human non-rapid-eye-movement (NREM) sleep. In addition to increasing locally in task-related areas, sleep-like slow waves also increase globally following sleep restriction or deprivation (27, 28). In animal models, these waves have been shown to reflect neuronal off-periods resembling those underlying the emergence of NREM slow waves (30). Crucially, sleep-like slow waves are associated with performance impairment and loss of task focus when occurring in task-related areas (28, 31). Given these observations, sleep-like slow waves during wakefulness have been suggested to represent a sign of neuronal (and cognitive) fatigue.

Interestingly, frontal brain areas involved in executive functions seem to be particularly vulnerable to fatigue and are among the first to show increases in the occurrence of local, sleep-like slow waves during extended wakefulness (28). In light of this, observations commonly attributed to the ego depletion phenomenon may actually reflect a form of task-dependent neural fatigue associated with sleep-like slow waves in frontal brain areas. Based on this view, the high variability in findings among previous ego-depletion studies could be due to the short duration of the tasks used in the literature, which may fail to induce sufficiently strong and consistent levels of functional fatigue in study participants.

In the present preregistered research, we investigated whether an extended period of practice with tasks requiring decision-making and self-control could lead to local sleep-like activity within frontal areas and alterations in socially relevant behaviors, as measured by EG. The employed tasks were selected from a previous work demonstrating their efficacy and specificity at inducing changes in self-control and frontal brain activity, in contrast to other tasks relying on different cognitive functions and brain areas (27, 28). Here, we used two versions of the same tasks requiring or not the exertion of executive functions, to obtain different levels of efficacy in inducing task-specific slow waves and behavioral impairment.

In contrast to previous studies in the literature, we used longer fatigue-inducing tasks (∼45 min vs. ∼10 min). Furthermore, we collected brain activity (high-density EEG) measures that allowed us to identify the connection between ego depletion manipulations and changes in sleep-like activity. Finally, a battery of one-shot, anonymous, and incentivized EG was administered to assess the potential effects of prolonged task practice on behavior. We conducted two studies on a total of 447 healthy adults who were assigned to one of two experimental conditions, the No Fatigue (NF) or Frontal Fatigue (FF) groups. Study 1 included both behavioral and EEG measures (*N* = 44) to assess changes in brain activity associated with the applied behavioral manipulation, while Study 2 replicated behavioral findings on a larger participant sample (*N* = 403).

## Results

### Delta and Theta EEG Activity.

**Fig. 1.**
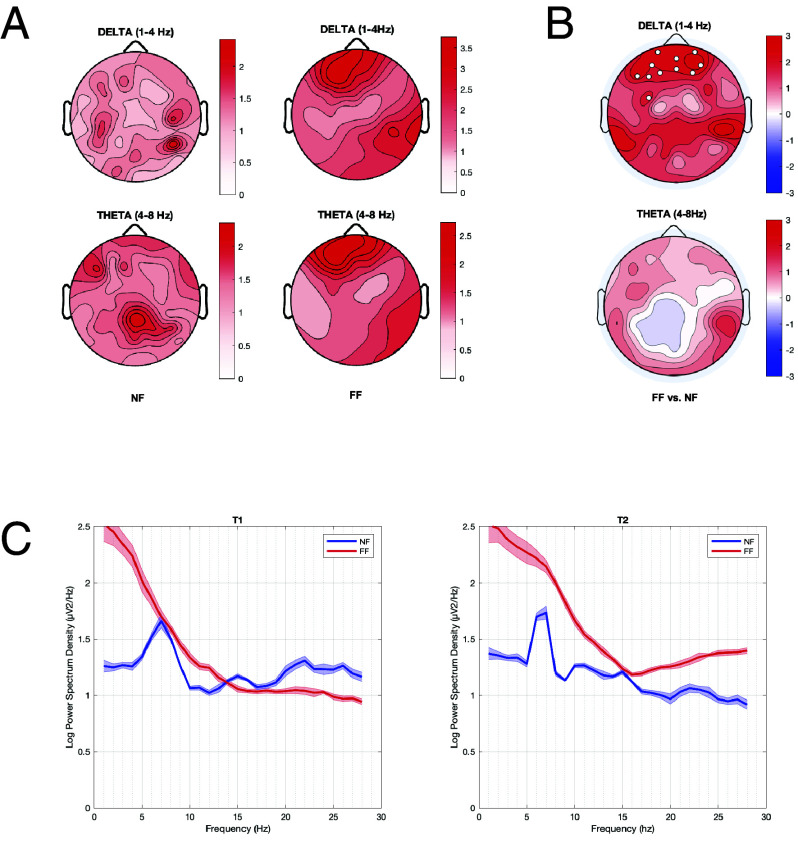
Changes in the low-frequency activity associated with the FF condition. Panel (*A*) shows low-frequency EEG activity changes associated with the FF and NF experimental conditions. Topographic plots on the *Left* show T0 to T1 changes (ratio) in delta (*Top*) and theta (*Bottom*) power (from 0 to a maximum of +3) for the two experimental conditions. In panel (*B*), the topographic plots show the statistical comparison between experimental conditions for the two frequency bands, with colormap indicating T-values (−3; +3). White dots mark *P* < 0.05, cluster-based correction. Panel (*C*) shows power spectral density (PSD) changes computed in the significant frontal cluster highlighted in Panels *A* and *B*. The *Left* plot shows the change in PSD in the NF group (blue) and in the FF group (red) after T1, while the *Right* plot shows the PSD difference after subjects performed the EG (T2).

**Fig. 2.**
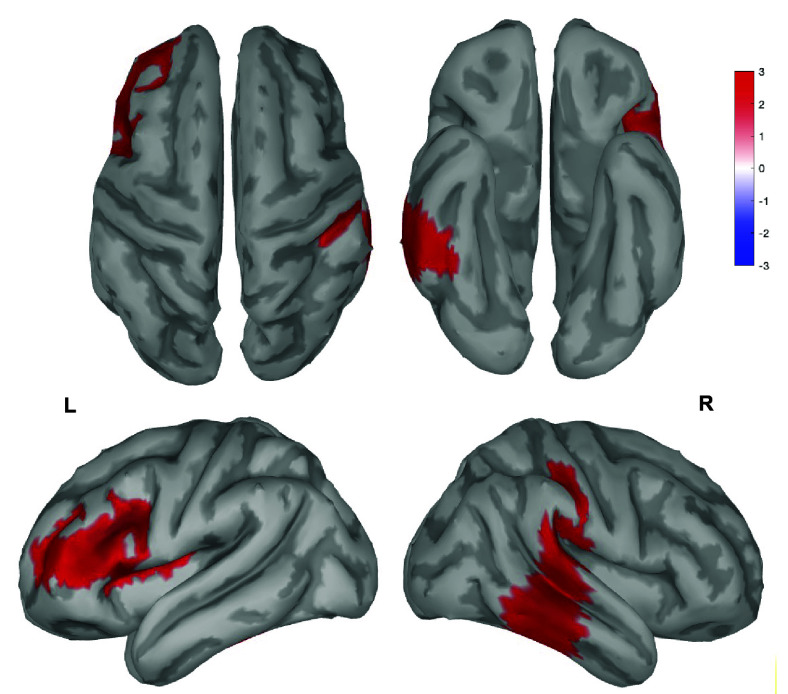
Source analysis of the delta EEG activity associated with the FF condition. Significant differences (*P* < 0.01, cluster-based correction) in T1/T0 delta ratios between FF and NF. Specifically, difference peaks were found in the left IFG (−36, 16, 28) and in the right middle temporal lobe (70, −30, −2).

To determine whether the administration of the fatigue-inducing paradigm would lead to increases in low-frequency (delta/theta) activity, we compared the relative change in signal power induced by the experimental procedures (from T0 to T1) across the two groups (Fig. 1*A*). The increase in delta activity (1 to 4 Hz) was significantly stronger in the FF group compared to the NF group in a frontal cluster of electrodes (Fig. 1*B*). Source localization showed that the delta power difference was maximal within the left inferior frontal gyrus (IFG) and in the right middle/superior temporal gyri (*P* < 0.01, cluster-based correction, Fig. 2). Instead, T0 to T1 theta activity (4 to 8 Hz) changes did not show significant differences across groups. However, a stronger increase in the FF relative to the NF condition was found after the EG in temporoparietal and occipital electrodes (T2, corrected *P* < 0.05, *SI Appendix*, Fig. S1). Source-modeling identified the peak of theta power increase in the right temporal lobe (*P* < 0.01, cluster-based correction, *SI Appendix*, Fig. S1).

### EG.

The following game decisions were collected: Dictator Game split, Ultimatum Game proposer offer, Ultimatum Game minimum acceptance threshold (MAT), contribution to the Public Goods Game without punishment, contribution to the Public Goods Game with punishment, and punishment decision in the Public Goods Game with punishment. Analyses were performed employing nonparametric tests and regression analyses. The specific test or model was selected depending on the nature of the dependent variable (binary, ordered, categorical, roughly continuous). Moreover, we performed individual tests for prosocial, antisocial, and spiteful modalities of punishment against the reference category of second-order free riding.

#### Study 1.

**Fig. 3.**
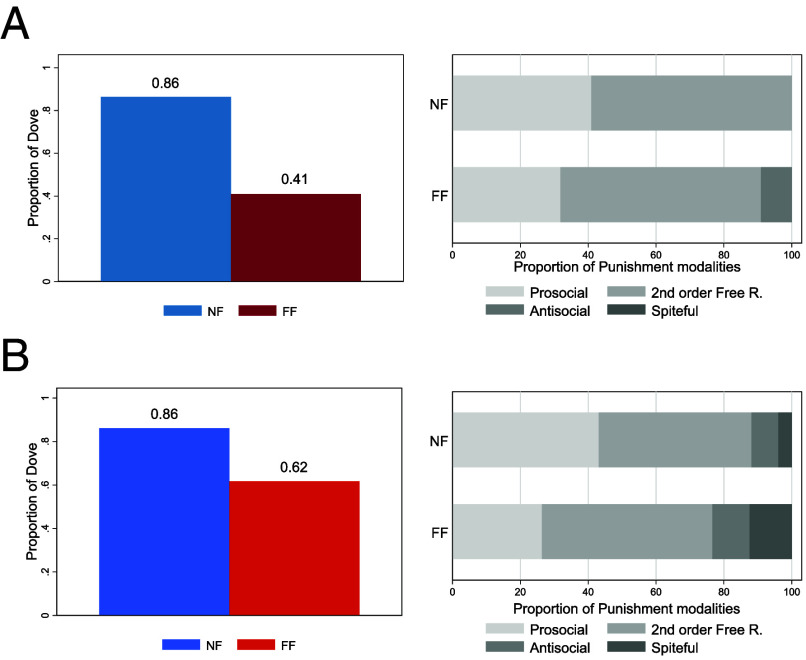
Results for the Hawk and Dove game and the punishment modalities. Panel (*A*) shows the results for the Hawk and Dove game and the punishment modalities for Study 1 (*N* = 44), while panel (*B*) shows the same results for Study 2 (*N* = 403). *Left* panels display the proportion of players who chose the peaceful option in the Hawk and Dove game. Players of the NF group (blue) chose the dove alternative significantly more with respect to players of the FF group (red). *Right* panels show the distribution of punishment modalities. In both studies, prosocial punishment and secon order-free riding are the two most represented modalities in the NF group. In the FF group, instead, antisocial and spiteful punishment are more represented, and in Study 2, a reduction in the selection of prosocial punishment is observable.

Fig. 3*A* shows the results of the Hawk and Dove Game and the punishment modalities in the Public Goods Game. The proportion of dovish behavior (i.e., peaceful cooperation) dropped from 86% to 41% (*P* = 0.0017, Pearson’s chi-squared test), which is consistent with a tendency of the FF group to engage more often in hawkish behavior (i.e., aggressive seizing of resources). This result was robust to the use of a probit model, *P* = 0.0020. Of note, an additional probit analysis showed that delta-power variations from T0 to T1 in frontal electrodes (see *SI Appendix*, Fig. S11 and *Supporting Methods*) were positively associated with the likelihood of playing aggressively in the Hawk and Dove game (*P* = 0.0408; SE clustered at the individual level). No other significant result was found in the rest of the games (*SI Appendix*, Fig. S3). We performed a total of ten tests. Hence, our results were robust to concerns regarding multiple testing and would survive any suitable correction. We did not find any results regarding differences in beliefs between groups (*SI Appendix*, Fig. S8), and a diff-in-diff analysis did not show differences in the average accuracy and response times of subjects in the Go/NoGo task (*SI Appendix*, Fig. S6).

#### Study 2.

Fig. 3*B* shows the results of the Hawk and Dove Game and the punishment modalities in the Public Goods Game. Consistent with the results of Study 1, behavioral choices significantly differed between the NF and FF groups (*P* < 0.001, Person’s chi-squared test). The result also survived the use of a probit model (*P* < 0.001). Concerning punishment, Pearson’s chi-squared test showed significant differences in the distribution of punishment modalities (*P* < 0.001). Specifically, pairwise Pearson chi-squared tests showed a significant reduction in prosocial punishment (*P* < 0.001) and a significant increase in spiteful punishment (*P* = 0.0019), while total punishment was unchanged (*P* = 0.2961). These results also survived the use of probit models (*P* < 0.001 and *P* = 0.0022, respectively) and a multinomial logit (*SI Appendix*, Table S10). No other significant result was found in the remaining games (*SI Appendix*, Fig. S4). Diff-in-diff regressions did not show any difference in subjects’ average accuracy and response times in the Go/NoGo task (*SI Appendix*, Fig. S6).

## Discussion

Moral precepts frequently compel individuals to engage in actions seemingly detrimental to their immediate self-interests, including core objectives like survival and reproduction. Yet, actions that benefit others have been suggested to enhance survival and reproduction through indirect means, such as integration into social groups and cultural societies (32–35). Nevertheless, fitness is also achieved through aggressive acts, which provide the individual with immediate benefits that are often able to outweigh the long-term consequences that such behaviors might hold (36), explaining why aggression is a basic form of human interaction (37).

Aggressive behavior plays a pivotal role in the animal kingdom, and research demonstrated that, in humans, it is modulated by distinct brain structures involved in emotion regulation and inhibitory control, and its impromptu expression depends on failures of self-regulation processes (38, 39). Consequently, self-control, functioning as the cognitive mechanism that facilitates the inhibition of instinctual responses in favor of actions endowed with a greater value, is intimately related to aggressive behavior and punishment activities.

Here, we demonstrated that the extended exertion of self-control leads to an increased propensity to engage in aggressive choices and a shift in punishment behavior. Furthermore, individuals who engaged in cognitive tasks requiring self-control presented an increase in sleep-like (delta) EEG activity within frontal areas involved in executive functions. Such a change is consistent with a use-dependent increase in local functional fatigue and may thus explain behavioral changes as reflecting the loss of efficient top–down executive regulation.

### Extended Exertion of Self-Control Affects Subsequent Socially Relevant Choices.

Previous research suggested that humans possess a finite reserve of self-regulatory resources, which may be depleted with extended use, a phenomenon defined as “ego depletion” (14). According to this view, experimental manipulations expected to induce ego depletion have been widely employed to investigate the role of self-control in socially relevant human behaviors. However, research produced inconsistent results. Indeed, some studies showed that when individuals experience cognitive fatigue due to prior self-regulation, they exhibit diminished empathy, reduced willingness to engage in prosocial acts, and a lowered threshold for frustration and aggression control in social interactions (18, 40, 41). Other studies, though, failed to detect any significant effects of ego depletion on social behavior (16, 42). Here, we propose that a potential explanation for those discrepancies could lie in the relatively short duration of typical ego depletion paradigms, which may be insufficient to induce measurable effects in most individuals. In line with our hypothesis, we found that about 45 min of practice with tasks requiring self-control led to consistent behavioral changes in two independent samples studied using similar experimental paradigms and procedures. Specifically, individuals who completed tasks requiring extended exertion of self-control showed a higher propensity to fight in the Hawk and Dove game relative to those who engaged in similar tasks not requiring self-control. Thus, when confronted with a situation in which agents can decide either to resolve a conflicting situation peacefully or aggressively, depleted individuals are more likely to choose the aggressive alternative. In addition, fatigued participants showed a lower probability of selecting the option regarding prosocial punishment, that is, the act of punishing defectors, and an increased propensity in choosing to punish spitefully, that is, punishing another player at random. Nonetheless, the overall propensity for punishment remained unchanged. Interestingly, prosocial punishment has been suggested to represent one of the potential mechanisms for the evolution of cooperation (43, 44). According to this idea, one might speculate that differences in the distribution of punishment modalities across different social groups of individuals (45) could be due to differences in vulnerability to task-dependent cognitive fatigue. However, studies about potential differences in the vulnerability to (frontal) local sleep across individuals or populations are currently missing. Overall, in line with previous research (46, 47), our results indicate that the tendency of individuals to engage in aggressive-like behaviors may increase following the extended use of self-control resources.

### Local Changes in Brain Fatigue and Sleep-Need May Underlie the Ego Depletion Effect.

A growing body of evidence demonstrated that contrary to long-held assumptions, sleep and wakefulness are not mutually exclusive—instead, they are locally regulated and may often coexist across distinct brain areas. During wakefulness, increases in the number and magnitude of local, sleep-like episodes characterized by slow (delta/theta) EEG waves have been reported following extended task practice and prolonged wakefulness with sleep restriction or deprivation (27–29, 48, 49). A local, use-dependent increase in the number of sleep-like episodes has been observed after prolonged practice with a variety of tasks, including self-control, visuospatial, and motor tasks (27–29). Importantly, sleep-like episodes appear to have a negative impact on behavioral performance when they occur during specific tasks and involve task-related brain areas (30). In light of these considerations, sleep-like episodes observed during wakefulness have been suggested to represent the signature of use-dependent neuronal fatigue and sleep needs (50). Therefore, we reasoned that frontal sleep-like episodes could offer a physiological explanation for behavioral changes observed after the exertion of self-control. Consistent with this, our present results showed that extended practice with tasks requiring self-control, but not practice with similar tasks not requiring self-control, is associated with a frontal increase in low-frequency, sleep-like EEG activity. A source modeling analysis confirmed that changes in sleep-like activity involved key areas strongly associated with the exertion of self-control, such as the left IFG and the right middle temporal cortex (51, 52).

Given these premises, we hypothesized that the effects attributed to ego depletion might actually depend on a use-dependent increase in the incidence of sleep-like episodes involving frontal brain areas associated with executive functions. Notably, though, the present findings did not allow us to demonstrate a direct causal link between sleep-like activity and decision-making, which should be the object of future research.

### Limitations of the Study.

Some limitations of the study should be acknowledged. The behavioral manipulation adopted in the present work was based on previous investigations showing that specific tasks induce changes in local brain activity and behavior consistent with self-control-related fatigue (28). However, we cannot entirely rule out the potential contribution of a general (non-task-specific) mental fatigue related to cognitive demands, as we did not include a control condition requiring similar cognitive efforts but distinct cognitive functions. Notably, though, no effects of the experimental condition (FF vs. NF) or interactions between condition and time (before vs. after task practice) were found for self-reported sleepiness, fatigue, mood, and positive/negative affects (*SI Appendix*, Table S2). A trend interaction was observed only for self-reported motivation but this effect did not survive correction for multiple testing (*P* < 0.05, uncorrected). Given these results, a nonspecific effect of fatigue appears unlikely to explain the behavioral differences observed in our study.

Moreover, while we tested several EG, we found clear significant effects of task-dependent fatigue only for the Hawk and Dove game. The reason why choices in the other games, such as the Dictator or Ultimatum games, were not affected by the behavioral manipulation is unclear and in partial contrast with previous observations (4, 11, 42). Nevertheless, our present results were robust to corrections accounting for multiple testing and were replicated across two independent samples. We suggest that choices in distinct EG may be differentially affected depending on the decision-making processes they reflect and the behavioral manipulation that is applied. The structure and complexity of the game may also play a role. For instance, the relative simplicity and clear-cut nature of the Hawk and Dove game may have facilitated the detection of cognitive alterations, which in contrast could be influenced by a variety of other mechanisms in more complex tasks (53).

Furthermore, while extensive research has been conducted on “short” ego depletion and repeated games (4), our experimental protocol adopted a longer depletion strategy and relied exclusively on one-shot games. Exploring the interplay between ego depletion resulting from extended exertion of self-control and repeated interactions could unravel whether, and to which extent, compensatory mechanisms could come into play to modulate individual choices that impact social life. Finally, the present study did not detect any differences in Go/NoGo performance across experimental conditions. We hypothesize that this could depend on the fact that a practice/learning period with the task was not included in the present study to reduce experimental demands. Therefore, learning and fatigue might have counteracted each other during the task, resulting in smaller and nonsignificant performance variation differences across experimental groups.

### Conclusions.

In conclusion, our study provides insights into the intricate relationship between self-control exertion, social behavior, and underlying neural mechanisms. We have demonstrated that extended exertion of self-control can lead to changes in neural activity within frontal cortical areas crucial for behavioral control and result in an increased propensity for aggressive choices in social interactions. This finding highlights the potential depletion of self-regulatory resources and its impact on prosocial behavior, shedding light on the inconsistent results reported to date in ego depletion research. Indeed, our results support the idea of ego depletion as a spectrum in which the strength of the effect depends on the intensity and/or duration of the prior manipulation, as well as the nature of the behavior being tested. Moreover, our investigation into local changes in brain fatigue and sleep-like activity offers a perspective on the ego depletion phenomenon. Thus, we propose that the increased sleep-like activity in frontal brain areas associated with executive functions and self-control underlie the observed behavioral changes. This use-dependent neuronal fatigue may not only provide a physiological explanation for the effects of ego depletion but, for extension, also of the previously described effects of sleep deprivation and restriction on societally impactful behaviors (54). The paradigm shifts from sleep as a global phenomenon to a locally regulated one occurred in relatively recent years, and its full range of implications for our understanding of human behavior is far from being exhaustively explored. While this perspective can offer a single and simple physiological explanation to a wide range of behavioral observations, future research will be necessary to understand the complexities of the interactions between local sleep regulation and other factors influencing human behavior.

## Materials and Methods

The experimental procedures adopted are briefly described in the following section. More details regarding ex ante power calculations to detect sample sizes, preregistration of both studies, prescreening, tasks description, EEG data analysis, and behavioral data analysis can be found in *SI Appendix*, *Supporting Methods*.

A total of 447 participants were recruited, with a final sample size of 44 subjects for Study 1 (22 females, mean age 30.3, SD of age 7.8) and 403 for Study 2 (251 females, mean age 23.3, SD of age 3.4). Participants were prescreened with an online questionnaire to exclude any medical, neurological, or psychiatric condition potentially affecting brain function and behavior. Study 1 protocol (No. 1485/2017) was approved by the Ethical Committee Area Vasta Nord Ovest, while Study 2 protocol (No. 03/2022) was approved by the Joint Ethical Committee for Research of the Scuola Normale Superiore and the Scuola Superiore Sant’Anna. All participants signed a written informed consent form before taking part in the studies and retained the faculty to drop at any time.

Prior to the beginning of the experimental sessions, participants were divided into identical matched groups, the FF group and the NF group, as detailed in *SI Appendix*, Table S1. Both groups completed experimental sessions with a similar structure and duration but involving partially different versions of the same tasks. Indeed, tasks presented in the FF condition required the exertion of self-control. In contrast, those in the NF condition did not require self-regulation and instead relied on more automatic processes.

The experimental procedures adopted for Studies 1 and 2 were identical, except for EEG measures exclusively performed in Study 1, as shown in *SI Appendix*, Fig. S7. Generally, an experimental session of Study 1 was structured as follows. First, the hd-EEG cap was applied to the participants (64 electrodes; EGI, Eugene, OR). Then, they completed a baseline test block lasting about 20 min and comprising a set of brief questionnaires, resting-state EEG activity (T0) recordings, and a computerized Go/NoGo task. Subsequently, participants completed three tasks requiring (or not) the exertion of self-control, such as an emotion suppression task (14), a false response task (28), and a classical Stroop task, each lasting approximately 15 min. Next, they completed another test block similar to the one presented at baseline (T1) and a set of EG lasting about 15 min. A final EEG resting-state recording (T2) was then performed, and the aim of the experimental procedure was explained to the participants. The day after the experimental session, all participants received an online set of self-report questionnaires by email to collect additional information regarding personality traits (e.g., empathy and aggressivity) and impulsiveness.

## Supplementary Material

Appendix 01 (PDF)

## Data Availability

Anonymized Excel Files have been deposited in Figshare (https://doi.org/10.6084/m9.figshare.25305496) (55).
