# Supplementary material for: Prolonged exertion of self-control causes increased sleep-like frontal brain activity and changes in aggressivity and punishment
Source: Proc Natl Acad Sci U S A. 2024 Nov 11;121(47):e2404213121. doi: 10.1073/pnas.2404213121 (PMC11588117; doi:10.1073/pnas.2404213121)
Supplement: Supplementary file 1 — Appendix 01 (PDF) [file pnas.2404213121.sapp.pdf]

Supporting Information for

Prolonged exertion of self-control causes increased sleep-like frontal brain activity and changes in aggressivity and punishment.

E. Ordali, P. Marcos-Prieto, G. Avvenuti, E. Ricciardi, L. Boncinelli, P. Pietrini, G. Bernardi and E. Bilancini

Erica Ordali, Pablo Marcos-Prieto
E-mail: erica.ordali@imtlucca.it, pablo.prieto@imtlucca.it

This PDF file includes:

Supporting Methods
Supporting Results
Figs. S1 to S11
Tables S1 to S9
SI References

Supporting Information Text

Supporting Methods

Ethics Approval. The whole study was conducted under two protocols defined in accordance with the ethical standards of the 2013 Declaration of Helsinki. Study 1 protocol (n.1485/2017) was approved by the Ethical Committee Area Vasta Nord Ovest, while Study 2 protocol (n.03/2022) was approved by the Joint Ethical Committee for Research of the Scuola Normale Superiore and the Scuola Superiore Sant'Anna. A fixed reimbursement (40*euros* for Study 1 and 10*euros* for Study 2) was provided to each participant upon completion of the experimental procedures. In addition, participants received a bonus payment relative to their performance in the incentivized economic games presented to them, as detailed below. The experimental procedures and expected results for both studies were pre-registered on AsPredicted (Study 1 and Study 2).

Participants. For Study 1, the sample size was determined according to a power calculation assuming a large target effect size (> 0.9). Of note, we had no previous literature from which we could derive a reliable estimate of the effect size to be expected in our investigation. Indeed, previous studies regarding the effects of typical ego-depletion paradigms employed relatively brief and simple behavioral manipulations, potentially unable to induce sufficiently strong levels of fatigue in most participants (1). On the other hand, previous studies investigating the effects of extended task practice and/or the relationship between sleep-like activity and behavior relied on relatively small sample sizes (2–5), but did not assess possible effects on decision-making processes in economic games. Therefore, we determined our minimum effect size of interest for Study 1 based on two main considerations. First, we planned to use an intensive fatigue induction procedure with a considerably longer duration with respect to traditional ego-depletion tasks. Second, we designed all experiments to ensure high levels of standardization and control to minimize the impact of potential confounding factors. Accordingly, we assumed a relatively large effect size could be expected. A power calculation with two independent samples, $\beta = 0.8$ and $\alpha = 0.05$, led to a minimum sample size of 21 subjects per group. A medium effect size was assumed for Study 2 as this investigation was performed in less controlled experimental conditions. In particular, we estimated that a sample size of 400 subjects would allow us to identify medium-size effects of around 0.25 for the continuous variables (one tail) using a non-parametric Wilcoxon-Mann-Whitney test with two groups.

Participants were recruited through flyers, social media, and by word of mouth within the Tuscany areas of Lucca and Pisa for Study 1 and Florence for Study 2. Potential volunteers were pre-screened with an online questionnaire to exclude any medical, neurological, or psychiatric condition potentially affecting brain function and behavior. We also assessed the presence of relevant sleep-related issues (Pittsburgh Sleep Quality Index – PSQI(6), cut-off score$<$10) and extreme chronotypes (Horne-Östberg Morningness-Eveningness Questionnaire – MEQ(7), scores ranging between 30 and 70). Individual preferences were measured in the pre-screening questionnaire through the "Global Preferences Survey", designed to assess a wide range of personal characteristics, such as time preferences (e.g., short-term or long-term orientation), risk preferences, positive and negative reciprocity, altruism, and trust(8). The admitted participants were subsequently contacted to schedule the experimental sessions. All volunteers signed a written informed consent form before taking part in the studies and retained the faculty to drop from the study at any time.

Together with biological sex, age, and level of education, the indexes derived from the Global Preferences Survey (positive reciprocity, negative reciprocity, altruism, patience, risk, trust) were used to divide participants into matched groups according to the mean and variance of the variables (see Table S1): the Frontal Fatigue group (FF) and the No Fatigue group (NF).

Structure of the Session. The experimental procedures are detailed in Figure S7. All experiments took place in the morning, from 9-10 AM to 12 PM, and involved a single participant at a time for Study 1 and up to 20 simultaneous participants for Study 2. The time window of each session was kept fixed to avoid possible confounding factors related to time-of-day effects or the influence of daily activities (e.g., work-related fatigue). Subjects were instructed to keep their usual sleep schedule the night before the experiment and to abstain from caffeinated beverages in the morning.

Additionally, the day after the experimental session, all participants received an online set of self-report questionnaires by email, measuring impulsivity-related behaviors and beliefs (Barratt Impulsiveness Scale - BIS-11(9)), individual tendencies toward aggressive behavior (Buss-Perry Aggression Questionnaire – BPAQ(10)) and empathy (Interpersonal Reactivity Index – IRI(11)). Questionnaires were sent to the subjects the day after the experiment to avoid potential confounds related to priming effects or effects related to the different experimental protocols (FF, NF).

Test Block.

Questionnaires. Participants were asked to self-report their mood, sleepiness, fatigue, and motivation using 10-point Likert scales and to complete the Positive Affect, Negative Affect Scale – PANAS(12). The scores obtained from the questionnaires were compared across experimental conditions to identify potential effects induced by the different cognitive engagements in FF and NF (SI Table S2).

Rest EEG Recordings. Resting-state EEG activity was recorded for 6 minutes (500 Hz sampling frequency), divided into three epochs of two minutes each. Recordings were performed three times: once in the baseline test block (T0), then at the test block after task practice and before the economic games (T1), and finally at the end of the experimental session (T2).

Go/NoGo Task. During test blocks T0 and T1, participants also completed two 5-minute trials of a classical Go/NoGo task(13–16), as in a previous work(2). Previous evidence indicated that extended exertion of self-control was associated with performance deterioration in this task. Here, we attempted to replicate this observation as a secondary aim of our study (see preregistration), as this would have been useful to demonstrate the efficacy of our experimental protocol in case no significant effects would emerge for the economic games. It is important to note, though, that our present study differs from previous investigations as a practice/learning session was not included to reduce experimental demands.

Task practice in FF and NF conditions . All participants completed three different tasks, each lasting about 15 minutes, for a total task practice time of $sim45$ minutes. In the FF condition, the tasks required high levels of impulse control, decision-making, and conflict resolution, while in the NF condition, a modified version requiring no exertion of self-control was presented(4). The three tasks were always presented in the same order and included an emotion suppression task(17), a false response task(2), and a classical Stroop task(18). A detailed description of the tasks is provided below. The computerized tasks used in Study 1 were implemented using E-Prime 2 (Psychology Software Tools, Pittsburgh, PA), while a PsychoPy implementation(19, 20) was used for Study 2.

Emotion Suppression Task. In the emotion suppression task(17, 21), participants watched a series of brief video clips showing humans and/or animals in amusing situations. Participants in the FF group were explicitly requested to completely suppress their facial reactions, whereas volunteers in the NF group were left free to express their emotional responses. Compliance with the task was assessed using a camera pointing at the participant's face in Study 1 or through direct monitoring by the experimenters of the participant's computer stations in Study 2.

False Response Task. During the false response task(2), study participants were presented with 180 simple short questions (e.g., *What is the color of a banana?*) and two possible responses (e.g., *Yellow or Blue*). After a 300 ms delay, a green or a red sign appeared below the question, indicating whether the participant had to provide the correct or wrong response, respectively. For participants in the FF group, the circle's color was randomly assigned for each stimulus, while it remained always green for participants in the NF group. Participants were required to respond as fast and accurately as possible. Adherence to the task requirements was verified through an a-posteriori assessment of task accuracy (50% chance level). Since some of the responses in Study 2 were not correctly recorded due to a technical issue, only the results from Study 1 are reported. Specifically, participants achieved accuracies of 60.3±18.3% (SD) and 89.9±6.2% in the FF and NF conditions, respectively ($p < 0.001$, vs. chance level, t-test).

Stroop Task. In the Stroop Task, participants were presented with color names written with the same ink color indicated by the name or a different color(18). Four trials, including two different versions of the task, were presented in alternating order: in one version, participants had to indicate the color name represented by the word (ignoring the ink color), while in the other version, they had to indicate the ink color (ignoring the color name). Each trial included 64 stimuli. Responses were provided using different response buttons associated with each of the four used colors, which were blue, red, yellow, and green. For participants in the FF group, the color name and the ink color could be either congruent (i.e., color ink and color name were matched) or incongruent (i.e., the color ink and the color name did not match), while for participants in the NF group, stimuli were always congruent (i.e., color ink and color name were always matched). Adherence to the task requirements was verified through an a-posteriori assessment of task accuracy (25% chance level). Since some of the responses in Study 2 were not correctly recorded due to a technical issue, only the results from Study 1 are reported. Specifically, participants achieved accuracies of 85.7±14.3% (SD) and 98.3±1.3% in the FF and NF conditions, respectively ($p < 0.001$, vs. chance level, t-test).

Economic Games. All subjects completed three blocks of economic games presented in random order: Block 1 included a Dictator Game followed by an Ultimatum Game, Block 2 included a Hawk and Dove Game, and Block 3 contained a Public Goods Game without Punishment, followed by a Public Goods Game with Punishment. Instructions for the games can be found in our online repository. Participants were informed that their counterparts in the economic games were other study participants and that the pairs or groups were changed for each game so that they would not interact with the same person twice. After each decision, participants were asked about their beliefs about others' average behavior for the whole experiment in the case of Study 1 or their own experimental session in the case of Study 2. One decision in the game and one belief were picked up at random at the end, and subjects were paid according to their earned points at the ratio of €1 for each experimental point, plus a bonus of two points if the chosen belief was close enough to the actual behavior. In addition to the fixed payoffs, participants received, on average, a game payoff of €13.17 in Study 1 and of €10.74 for Study 2. The economic games used in these experiments were implemented using oTree(22).

Dictator Game. Participants were matched in pairs and received the role of either dictator or recipient. The dictator had to decide how to split 20 points between himself and the recipient. The recipient has no action to take and just obtains the points decided by the dictator. Participants could earn two bonus points if they guessed the average split with an accuracy within one point of the actual value.

Ultimatum Game. Participants were matched in pairs and received the role of either proposer or responder. The proposer had to make an offer regarding how to split 20 points between himself and the responder. The responder was given the choice of accepting or rejecting offers. In case the responder accepts the offer, the proposed distribution is implemented. In case

the responder rejects the offer, both the proposer and the responder obtain zero points. The responder stated the minimum acceptance threshold that he was willing to accept, as done using the strategy method(23). Participants could earn two bonus points if they guessed the average proposal with an accuracy within one point of the actual value. Moreover, participants could earn two bonus points if they guessed the average minimum acceptance threshold with an accuracy within one point of the actual value.

Hawk and Dove Game. Participants were matched in pairs and were both given the option of taking action either as Hawk or Dove. When both players chose Dove, they both received 10 points. When one player chose Hawk and the other chose Dove, the Hawk received 15 points, while the Dove received 5 points. If both participants chose Hawk, both received zero points. Actions were labeled as A and B. In Study 1, the action Dove was always action A, while in Study 2, the label was randomly assigned. Participants could earn two bonus points if they guessed the proportion of Dove plays with accuracy within five percentage points of the actual value.

Public Goods Game without Punishment. Participants were matched in groups of four. Each participant received a personal endowment of 10 points and was given the option to put any amount in a common pool, keeping the rest. Contributions to the common pool were then doubled and divided equally between all members of the group. Participants could earn two bonus points if they guessed the average minimum contribution with accuracy within half a point of the actual value.

Public Goods Game with Punishment. The procedure was similar to the Public Goods Game without punishment. However, in this case, participants were given the option to spend two points to reduce the earnings of another player by four points. This decision was presented using the strategy method to reduce feedback negativity. Therefore, participants decided, without receiving any feedback, whether they would like to punish the person who contributed the most ("antisocial punishment"), the person who contributed the least ("prosocial punishment"), one person at random ("spiteful punishment"), or not punish at all ("second-order free riding"). Participants could earn two bonus points if they guessed the average minimum contribution with accuracy within half a point of the actual value. Moreover, they earned two bonus points if they guessed both the most and least frequent modalities of punishment.

EEG data analysis. The resting-state EEG recordings were band-pass filtered between 0.5 and 45 Hz (Kaiser-windowed finite impulse response filter; −0.01 dB passband gain, −40 dB stopband gain, 0.49 Hz roll-off) and divided into non-overlapping 4-second epochs(4). The resulting traces were visually inspected to identify and mark bad channels and epochs containing clear artifactual activity. Then, an independent component analysis (ICA) was performed in EEGLAB(24) to remove signal components reflecting ocular, muscular, and electrocardiograph artifacts. Rejected bad channels were subsequently interpolated using spherical splines. Finally, for each EEG derivation, the signal was average-referenced. Power spectral density (PSD) estimates were computed using Welch's method (pwelch function) in each 4-second epoch (2-s Hamming windows, 50% overlap). Finally, as delta (0.5-4 Hz) and theta (4-8 Hz) frequency bands are knowingly associated with sleep states, signal power was computed for those bands and averaged across epochs.

Source-modeling of EEG data. Source modeling of EEG data was performed using Brainstorm(25). Specifically, the conductive head volume was modeled using a three-layer symmetric boundary element method (OpenMEEG BEM(26)) and the default ICBM152 anatomical template. The forward model was constructed using a standard set of electrode positions (GSN HydroCel 64). The source space was constrained to the cerebral cortex, which was modeled as a three-dimensional grid of 15,002 vertices. The inverse matrix was computed using the standardized low-resolution brain electromagnetic tomography (sLORETA) constraint with a regularization parameter equal to $10^{-2}\lambda$. Finally, the signal power was computed for each vertex in the source space using Welch's method (1-s Hamming windows, 50% overlap).

Scalp and source level analyses of EEG data. Comparisons of EEG signal power across experimental conditions were performed using paired t-tests and a permutation-based cluster-mass correction(27). In brief, each contrast was repeated (10,000 permutations) after shuffling the labels of the experimental conditions, and all clusters of significant electrodes were identified ($p < 0.05$). Then, we computed the sum of test statistics across electrodes belonging to the same cluster, and the maximum obtained value was saved in a frequency table. A minimum cluster-mass threshold corresponding to the 95th percentile of the resulting distribution was applied to correct for multiple comparisons. For comparisons performed at the scalp level, the analyses were restricted to 51 "internal" electrodes in order to minimize the possible impact of residual artifactual activity in channels located near the eyes or on the temporal/neck muscles(28). All statistical analyses were performed in MATLAB. At the source level, power maps were exported to MATLAB, and statistical comparisons were performed as described for scalp analyses. The obtained results were re-imported in Brainstorm for visualization.

Analysis Models. Supplementary Information Tables from S3 to S9 show regression analyses for each game using the pooled database in which three groups of control variables are added. Specifically, the demographic variables group includes age, gender, and education; the preference variables group includes our proxies of positive reciprocity, negative reciprocity, altruism, patience, risk, and trust; and the questionnaire variables group includes the Interpersonal Reactivity Index, Barratt's Impulsiveness Scale, and the Buss-Perry Aggression Questionnaire. Specifically, for the Hawk and Dove Game choice, probit models were used. For the split in the Dictator Game, the offer and the minimum acceptance threshold in the Ultimatum Game, and the contributions in the Public Goods Game with and without Punishment, tobit models were used. Finally, for the Punishment

modalities in the Public Goods Game, probit models were used for the individual categories and a multinomial logit for the combined category.

Additional Analysis. Thanks to the suggestions of an anonymous reviewer, we performed an additional analysis for better investigating and verifying the association between brain activity and decision-making changes. Therefore, we performed a new analysis as described here. First, for each participant and EEG recording, we computed the mean delta power (1-4 Hz) across frontal electrodes (see topographic map in Figure S11). Then, we computed the average across T1 and T2, and the resulting values were used to compute the ratio with respect to baseline power levels (T0). Finally, we performed a regression analysis using all participants of Study 1 ($N = 44$) to predict behavioral results based on delta-power variations in frontal electrodes, clustering standard errors at the individual level. Our probit analysis showed a positive association between increases in frontal delta power and the likelihood of playing aggressively in the Hawk and Dove game ($p = 0.0408$).

Tables information

The tables refer to the whole sample between the two experiments (pooled data, N=447). Analysis of each experiment separately and analysis of each separate modality of punishment are available in our online repository.

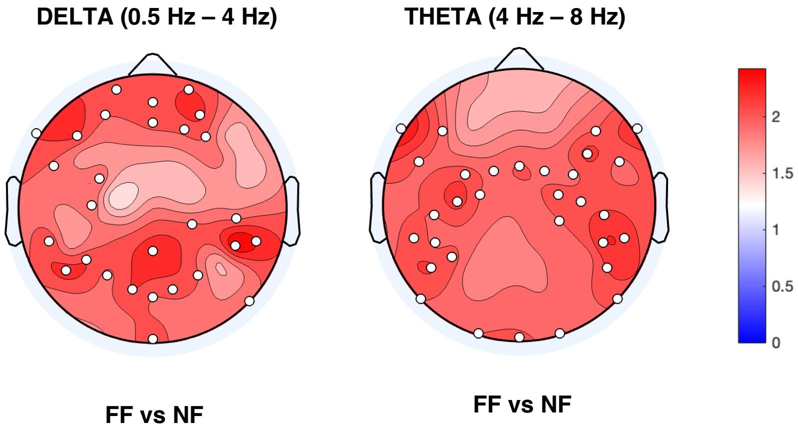

Fig. S1. Differences in low-frequency activity variations after T2. The topographic plots show the statistical comparison between experimental conditions (FF vs NF) for delta and theta frequency bands at time T2 after subjects completed the economic games block. Colormap indicates T-values between +3 and -3, while white dots mark electrodes significant at $p < 0.05$ (cluster-based correction).

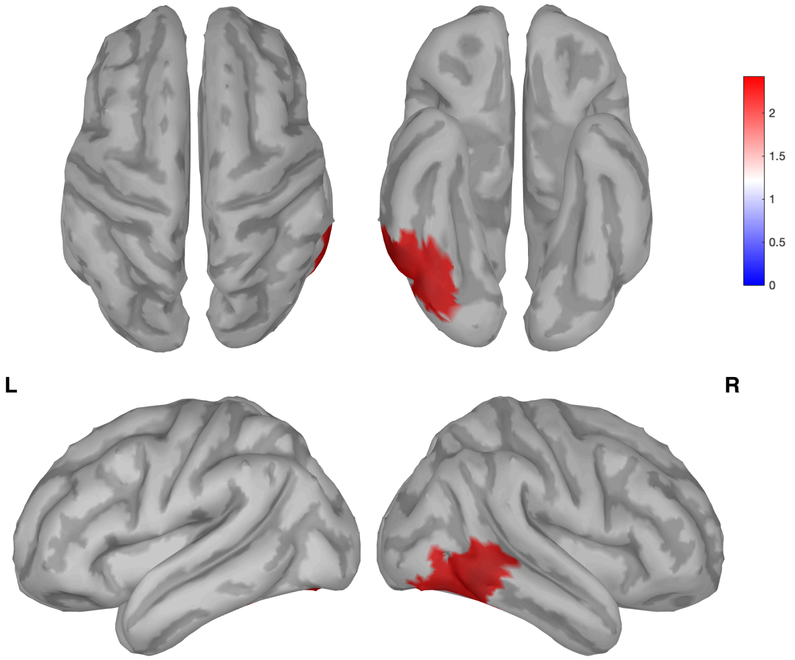

Fig. S2. Source analysis of the theta EEG activity associated with the FF condition in T2. Significant differences ($p < 0.01$, cluster-based correction) in T2/T0 theta ratios between FF and NF. The difference peak was found in the right temporal lobe (64, -54, -10).

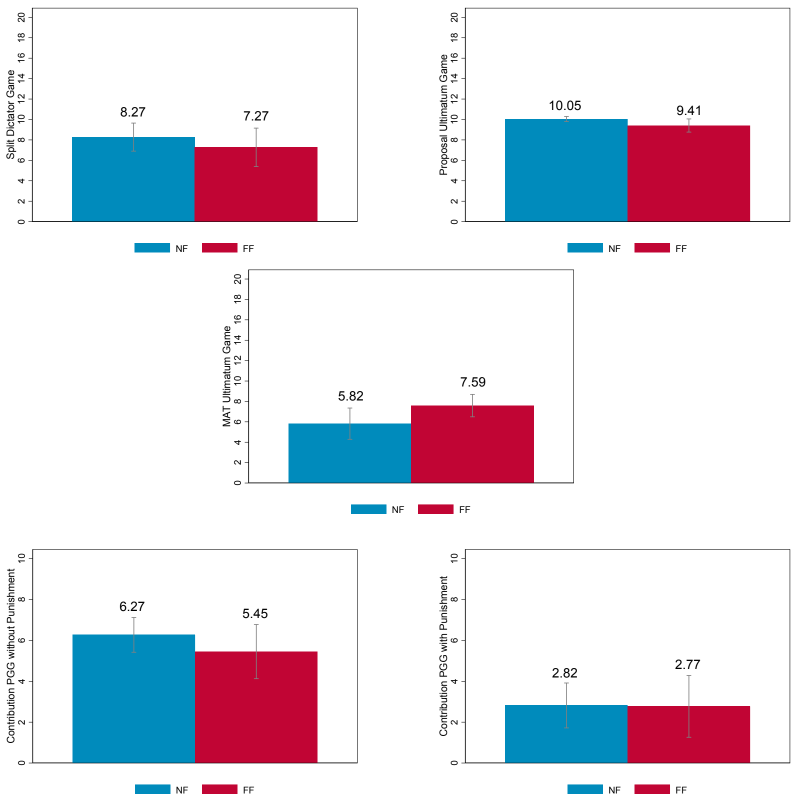

Fig. S3. Results of the other games of Study 1. Plots are presented in the following order from left to right and top to bottom: split of the Dictator Game, proposition in the Ultimatum Game, minimum acceptance threshold for the Receiver of the Ultimatum Game, and contribution in the PGG without and with punishment.

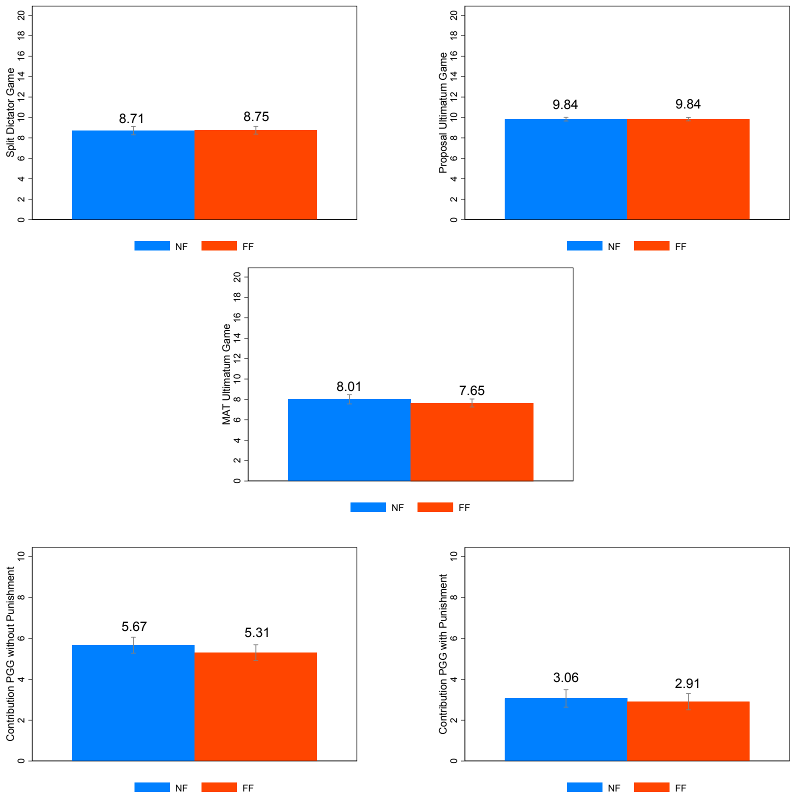

Fig. S4. Results of the other games of Study 2. Plots are presented in the following order from left to right and top to bottom order: split of the Dictator Game, proposition in the Ultimatum Game, minimum acceptance threshold for the Receiver of the Ultimatum Game, contribution in the PGG without and with punishment.

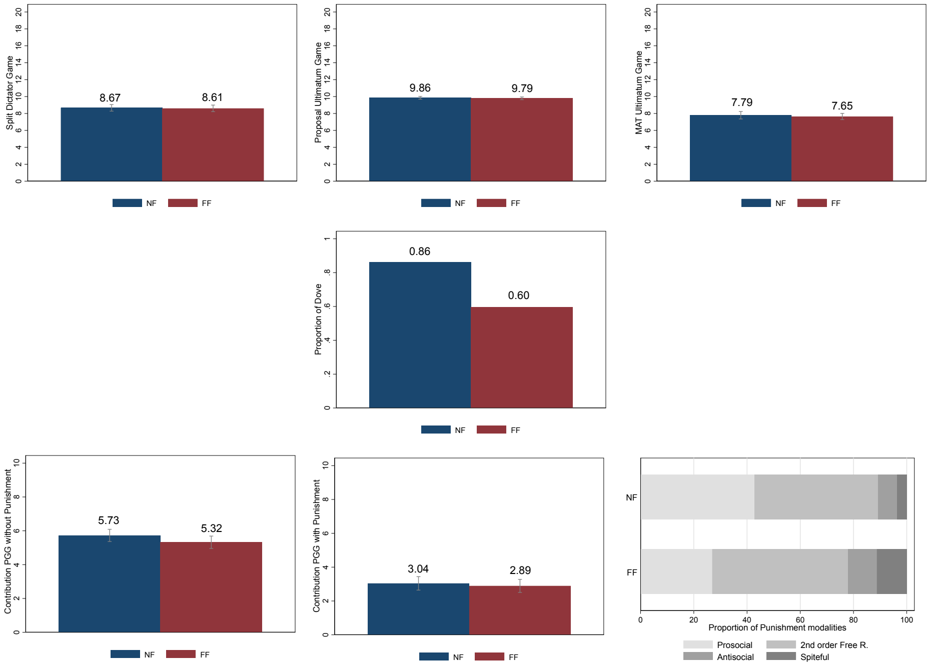

Fig. S5. Results of the games (pooled data). Plots are presented in the following order from left to right and top to bottom order: split of the Dictator Game, proposition in the Ultimatum Game, minimum acceptance threshold for the Receiver of the Ultimatum Game, contribution in the PGG without and with punishment. The last plot shows the proportion of punishment modalities.

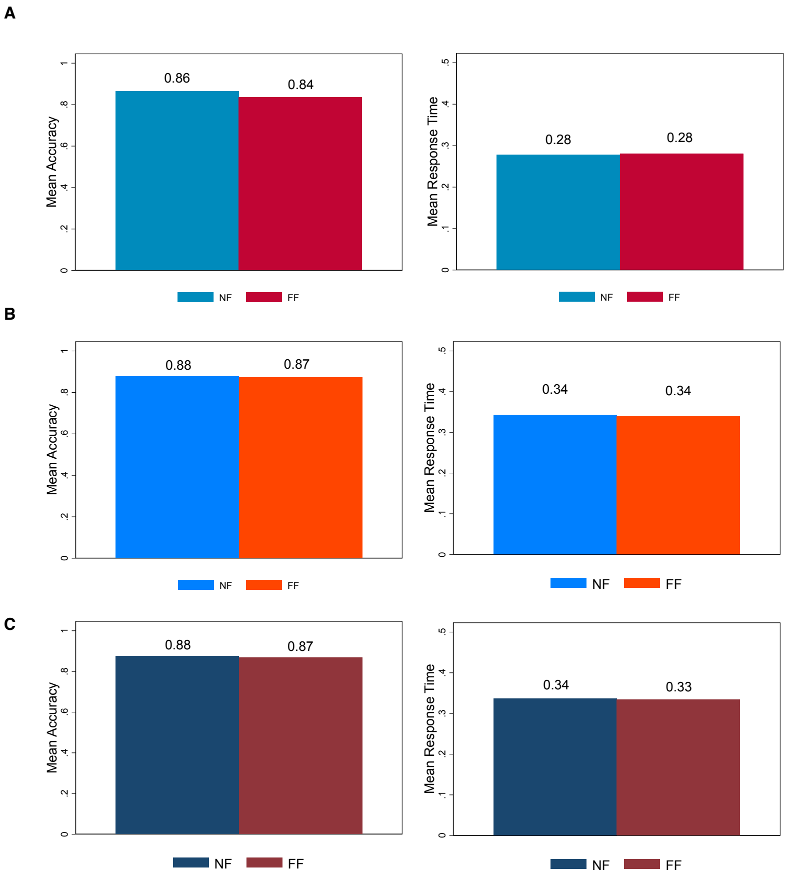

Fig. S6. Go/NoGo analysis. Results of mean accuracy and mean reaction times for the Go/NoGo Task in the two experiments. Panel A shows the results of Study 1, and panel B the ones of Study 2. Finally, panel C shows the results of the two studies merged.

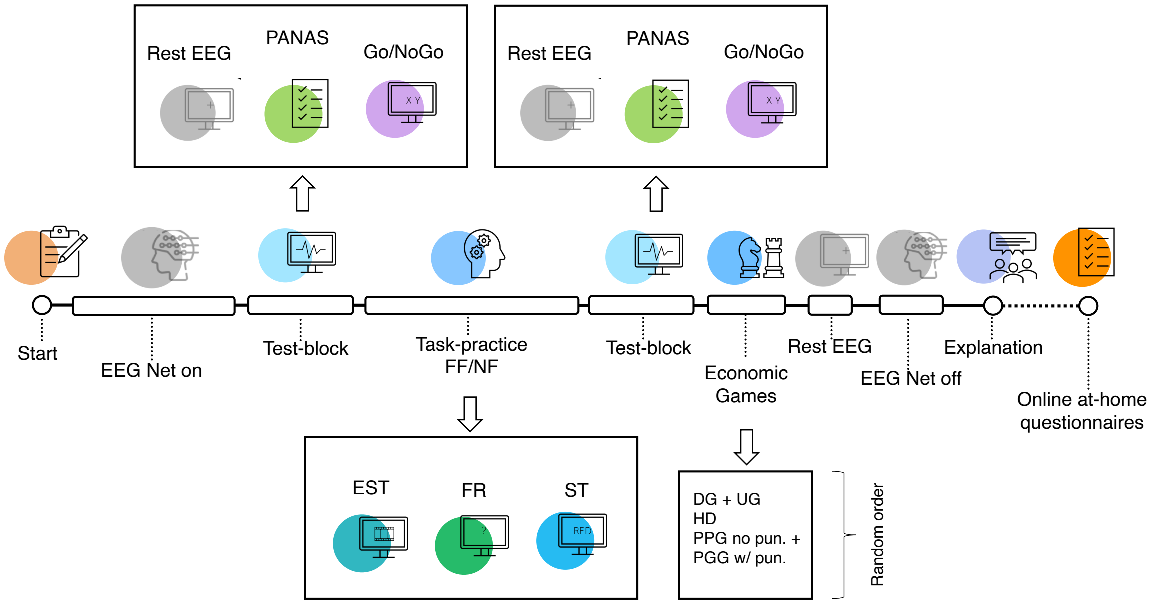

Fig. S7. Visual articulation of an experimental session. Gray icons represent EEG-related operations present in Study 1 but not in Study 2. The length of the white bars reflects the relative length of each phase performed in the lab.

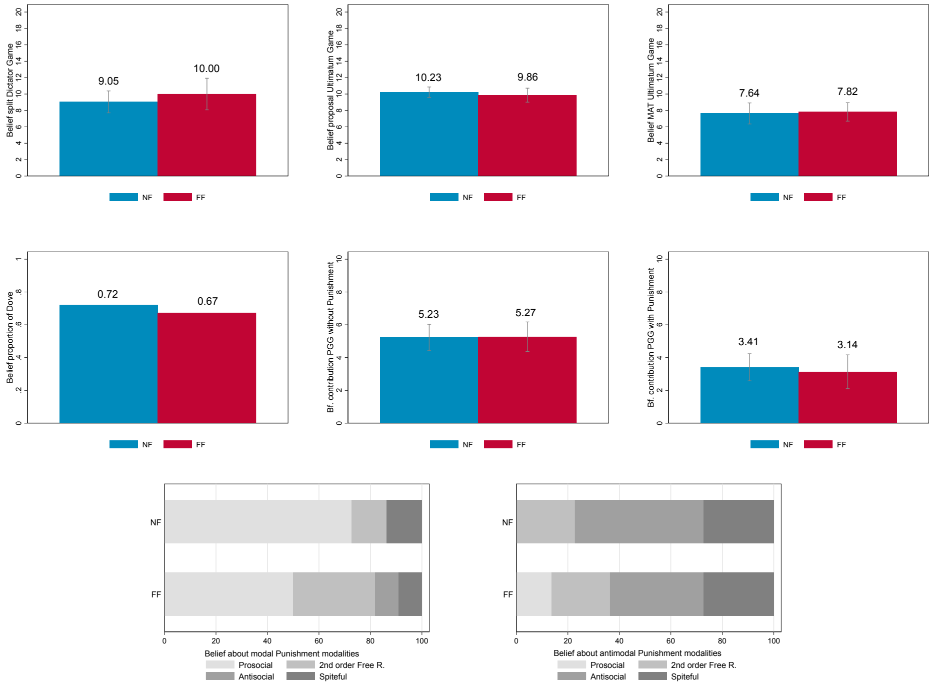

Fig. S8. Results for the stated beliefs in Study 1. The order of the panels is the following: Dictator Game (average split), Ultimatum Game (average split and minimum acceptance threshold - MAT), Hawk and Dove Game (proportion of people that played “dove”), Public Goods Game without punishment (average contribution), Public Goods Game with Punishment (average contribution, most frequent and infrequent punishing behaviors). No significant results were found.

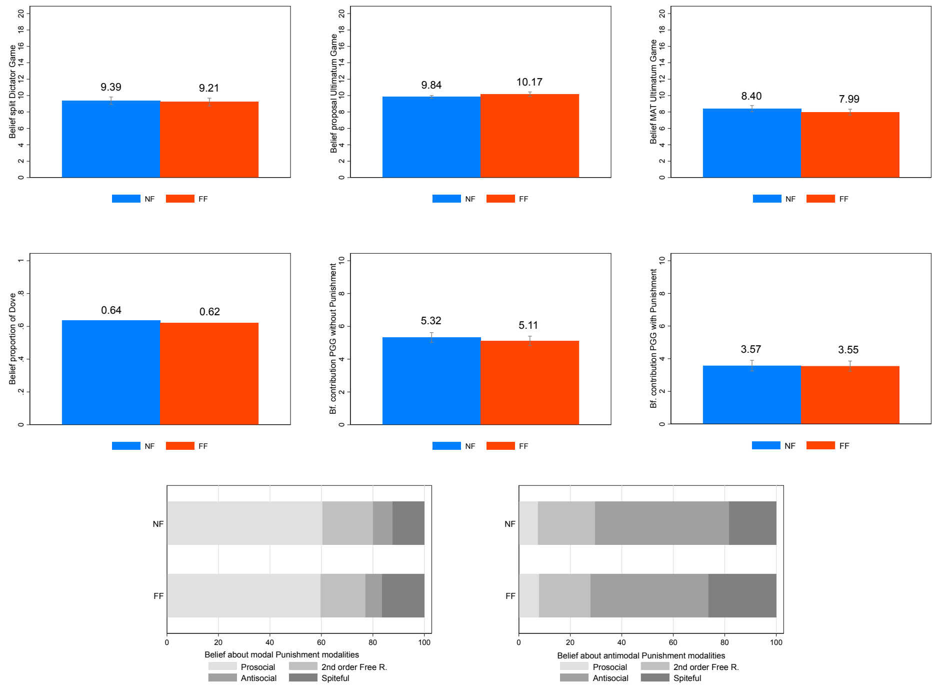

Fig. S9. Results for the stated beliefs in Study 2. The order of the panels is the following: Dictator Game (average split), Ultimatum Game (average split and minimum acceptance threshold - MAT), Hawk and Dove Game (proportion of people that played "dove"), Public Goods Game without punishment (average contribution), Public Goods Game with Punishment (average contribution, most frequent and infrequent punishing behaviors). A marginally significant result was found in the subjects' beliefs about the minimal proposal in the Ultimatum Game (MAT, $p = 0.0444$) but this effect did not survive after pooling the data across studies (see Figure S10).

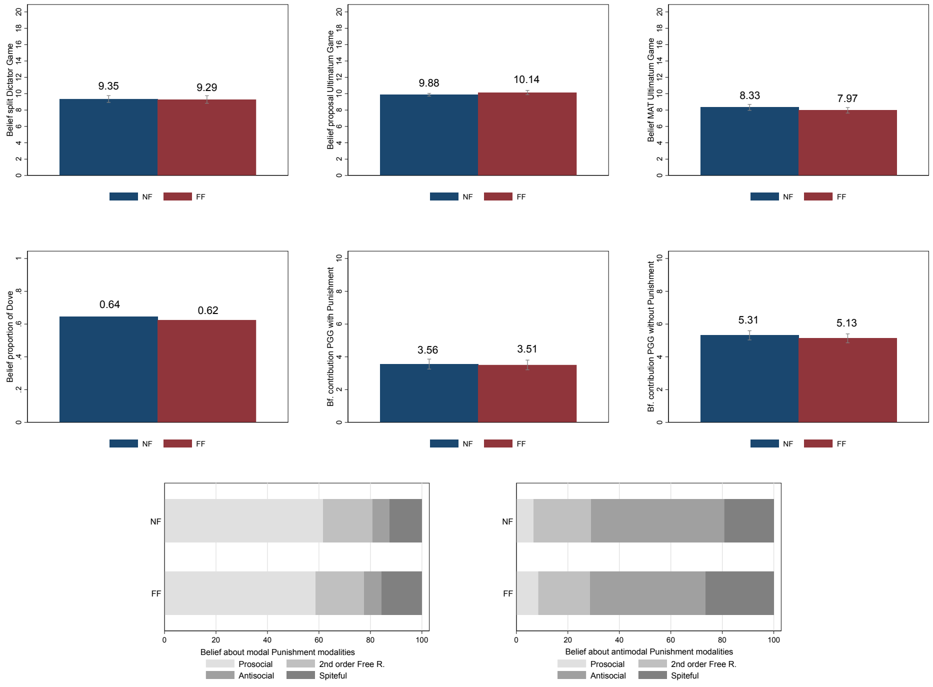

Fig. S10. Results for the stated beliefs obtained after pooling the data of the two experiments. The order of the panels is the following: Dictator Game (average split), Ultimatum Game (average split and minimum acceptance threshold - MAT), Hawk and Dove Game (proportion of people that played “dove”), Public Goods Game without punishment (average contribution), Public Goods Game with Punishment (average contribution, most frequent and infrequent punishing behaviors). No significant results were found.

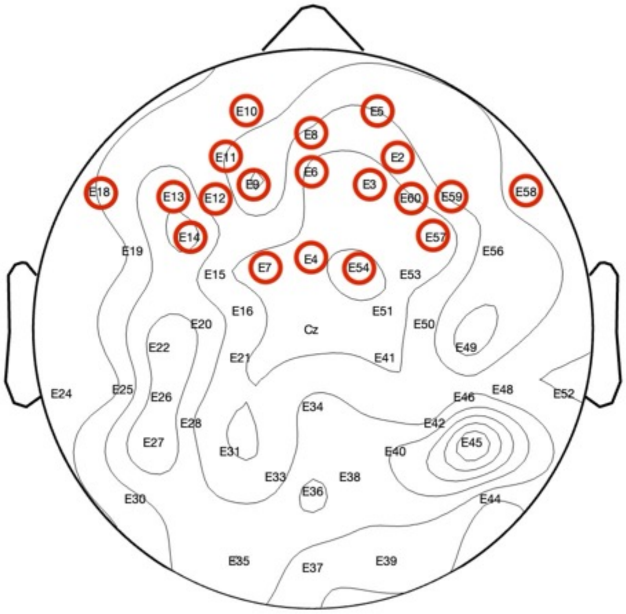

Fig. S11. Cortical map of the electrodes used (red circles) to compute the additional analysis.

Table S1. Matching of participants

	Means			St. Deviation		
	No Fatigue	Fatigue	p t-test	No Fatigue	Fatigue	p F-test
Age	23.9777	24.0090	0.9416	4.4444	4.5806	0.6529
Gender	0.6161	0.6054	0.8172	0.4874	0.4899	0.9407
Education	13.9821	13.9283	0.7360	1.6511	1.7253	0.5122
Pos. Reciprocity	0.0163753	-0.0166556	0.643	0.7482413	0.7572825	0.8579
Neg. Reciprocity	0.0002061	0.0004495	0.9975	0.8367353	0.8085697	0.6108
Altruism	-0.0307196	0.0328269	0.404	0.8155425	0.7910178	0.6496
Patience	-0.002936	0.0027184	0.9408	0.8469781	0.7597402	0.1057
Risk	-0.0091699	0.009211	0.7997	0.7422482	0.7875032	0.3779
Trust	-0.0035312	0.0035631	0.8964	0.5940963	0.5547887	0.3086

The table shows matched characteristics of the two groups, which included biological sex, age, level of education, and the indexes derived from the Global Preferences Survey (pooled data). For these latter indices, standardized values are reported instead of raw values. Results about means are robust to the Wilcoxon rank-sum tests and results about standard deviations are robust to Levene's robust test statistic and Brown's and Forsythe robust test statistics.

Table S2. Likert Scales - tobit estimates

	(1) Sleepiness	(2) Mood	(3) Fatigue	(4) Motivation	(5) Pos. affects	(6) Neg. affects
Condition	-0.127	-0.137	-0.189	-1.454*	-0.122	0.0827*
	[0.273]	[0.632]	[0.232]	[0.614]	[0.0672]	[0.0405]
Time	1.003***	-0.531	2.020***	-1.915**	-0.401***	-0.0968*
	[0.287]	[0.616]	[0.254]	[0.605]	[0.0670]	[0.0404]
Interaction	-0.349	-0.262	0.663	1.948*	0.110	-0.00474
	[0.399]	[0.847]	[0.359]	[0.791]	[0.0948]	[0.0571]
Demographics	YES	YES	YES	YES	YES	YES
Preferences	YES	YES	YES	YES	YES	YES
Questionnaires	YES	YES	YES	YES	YES	YES
Constant	0.233	13.00***	0.959	4.862	4.055***	0.0144
	[1.643]	[3.612]	[1.439]	[3.080]	[0.386]	[0.233]
Observations	886	886	886	886	886	886

* $p < 0.05$, ** $p < 0.01$, *** $p < 0.001$

Standard errors in brackets.

The table displays diff-in-diff tobit estimates for the Likert Scales compiled by participants during the experiment (pooled data). The demographics group variable includes age, education, and biological sex. The preferences group variable includes Falk's et al. (2018) Global Preferences survey proxies for altruism, positive reciprocity, negative reciprocity, trust, patience, and risk aversion. Finally, the questionnaire group variable includes the Buss-Perry Aggression Questionnaire, the Interpersonal Reactivity Index, and Barratt's Impulsiveness Scale. Results are not corrected for multiple comparisons.

Table S3. Dictator Game splits - tobit estimates

	(1) Split DG	(2) Split DG	(3) Split DG	(4) Split DG
Condition	-0.0562	-0.0540	-0.0887	-0.0707
	[0.302]	[0.299]	[0.297]	[0.296]
Demographics	NO	YES	YES	YES
Preferences	NO	NO	YES	YES
Questionnaires	NO	NO	NO	YES
Constant	8.596***	9.908***	10.63***	9.567***
	[0.213]	[1.326]	[1.327]	[2.355]
Observations	447	447	443	443

* $p < 0.05$, ** $p < 0.01$, *** $p < 0.001$

Standard errors in brackets.

The table displays tobit estimates for the dictator's split in the Dictator Game (pooled data). The demographics group variable includes age, education, and biological sex. The preferences group variable includes Falk's et al. (2018) Global Preferences survey proxies for altruism, positive reciprocity, negative reciprocity, trust, patience, and risk aversion. Finally, the questionnaire group variable includes the Buss-Perry Aggression Questionnaire, the Interpersonal Reactivity Index, and Barratt's Impulsiveness Scale.

Table S4. Ultimatum Game proposal - tobit estimates

	(1) Proposal UG	(2) Proposal UG	(3) Proposal UG	(4) Proposal UG
Condition	-0.0634	-0.0588	-0.0787	-0.0757
	[0.119]	[0.118]	[0.116]	[0.116]
Demographics	NO	YES	YES	YES
Preferences	NO	NO	YES	YES
Questionnaires	NO	NO	NO	YES
Constant	9.857^{***}	9.301^{***}	9.485^{***}	8.339^{***}
	[0.0841]	[0.523]	[0.519]	[0.923]
Observations	447	447	443	443

* $p < 0.05$, ** $p < 0.01$, *** $p < 0.001$

Standard errors in brackets.

The table displays tobit estimates for the sender's proposal in the Ultimatum Game (pooled data). The demographics group variable includes age, education, and biological sex. The preferences group variable includes Falk's et al. (2018) Global Preferences survey proxies for altruism, positive reciprocity, negative reciprocity, trust, patience, and risk aversion. Finally, the questionnaire group variable includes the Buss-Perry Aggression Questionnaire, the Interpersonal Reactivity Index, and Barratt's Impulsiveness Scale.

Table S5. Ultimatum Game MAT - tobit estimates

	(1)<br>MAT UG	(2)<br>MAT UG	(3)<br>MAT UG	(4)<br>MAT UG
Condition	-0.147	-0.138	-0.138	-0.153
	[0.301]	[0.297]	[0.299]	[0.298]
Demographics	NO	YES	YES	YES
Preferences	NO	NO	YES	YES
Questionnaires	NO	NO	NO	YES
Constant	7.769***	8.382***	8.676***	8.218***
	[0.213]	[1.316]	[1.334]	[2.369]
Observations	447	447	443	443

* $p < 0.05$, ** $p < 0.01$, *** $p < 0.001$

Standard errors in brackets.

The table displays tobit estimates for the receiver's minimum acceptance threshold (MAT) in the Ultimatum Game (pooled data). The demographics group variable includes age, education, and biological sex. The preferences group variable includes Falk's et al. (2018) Global Preferences survey proxies for altruism, positive reciprocity, negative reciprocity, trust, patience, and risk aversion. Finally, the questionnaire group variable includes the Buss-Perry Aggression Questionnaire, the Interpersonal Reactivity Index, and Barratt's Impulsiveness Scale.

Table S6. Hawk and Dove Game - probit estimates

	(1)<br>Play Dove	(2)<br>Play Dove	(3)<br>Play Dove	(4)<br>Play Dove
Condition	-0.843***	-0.849***	-0.877***	-0.889***
	[0.135]	[0.136]	[0.139]	[0.139]
Demographics	NO	YES	YES	YES
Preferences	NO	NO	YES	YES
Questionnaires	NO	NO	NO	YES
Constant	1.088***	0.860	1.090	1.945
	[0.104]	[0.579]	[0.596]	[1.091]
Observations	447	447	443	443

* $p < 0.05$, ** $p < 0.01$, *** $p < 0.001$

Standard errors in brackets.

The table displays probit estimates for the "dove" action of the Hawk and Dove Game (pooled data). The demographics group variable includes age, education, and biological sex. The preferences group variable includes Falk's et al. (2018) Global Preferences survey proxies for altruism, positive reciprocity, negative reciprocity, trust, patience, and risk aversion. Finally, the questionnaire group variable includes the Buss-Perry Aggression Questionnaire, the Interpersonal Reactivity Index, and Barratt's Impulsiveness Scale.

Table S7. Public Goods Game without punishment contribution - tobit estimates

	(1) Contribution	(2) Contribution	(3) Contribution	(4) Contribution
Condition	-0.433	-0.434	-0.443	-0.432
	[0.323]	[0.323]	[0.320]	[0.319]
Demographics	NO	YES	YES	YES
Preferences	NO	NO	YES	YES
Questionnaires	NO	NO	NO	YES
Constant	5.916***	5.084***	5.508***	5.190*
	[0.229]	[1.427]	[1.426]	[0.862]
Observations	447	447	443	443

* $p < 0.05$, ** $p < 0.01$, *** $p < 0.001$

Standard errors in brackets.

The table displays tobit estimates for contribution to the Public Goods Game without punishment (pooled data). The demographics group variable includes age, education, and biological sex. The preferences group variable includes Falk's et al. (2018) Global Preferences survey proxies for altruism, positive reciprocity, negative reciprocity, trust, patience, and risk aversion. Finally, the questionnaire group variable includes the Buss-Perry Aggression Questionnaire, the Interpersonal Reactivity Index, and Barratt's Impulsiveness Scale.

Table S8. Public Goods Game with punishment contribution - tobit estimates

	(1)<br>Contribution	(2)<br>Contribution	(3)<br>Contribution	(4)<br>Contribution
Condition	-0.293	-0.275	-0.260	-0.250
	[0.436]	[0.432]	[0.433]	[0.432]
Demographics	NO	YES	YES	YES
Preferences	NO	NO	YES	YES
Questionnaires	NO	NO	NO	YES
Constant	2.327***	1.853	2.056	-1.048
	[0.310]	[1.928]	[1.951]	[3.437]
Observations	447	447	443	443

* $p < 0.05$, ** $p < 0.01$, *** $p < 0.001$

Standard errors in brackets.

The table displays tobit estimates for the contributions to the Public Goods Game with a punishment option (pooled data). The demographics group variable includes age, education, and biological sex. The preferences group variable includes Falk's et al. (2018) Global Preferences survey proxies for altruism, positive reciprocity, negative reciprocity, trust, patience, and risk aversion. Finally, the questionnaire group variable includes the Buss-Perry Aggression Questionnaire, the Interpersonal Reactivity Index, and Barratt's Impulsiveness Scale.

Table S9. Punishment modalities - multinomial logit estimates

	Free Rider	Prosocial	Antisocial	Spiteful
Free Rider				
(base outcome)				
Prosocial punisher				
Condition	-0.562**	-0.559**	-0.579**	-0.577**
	[0.213]	[0.214]	[0.219]	[0.220]
Demographics	NO	YES	YES	YES
Preferences	NO	NO	YES	YES
Questionnaires	NO	NO	NO	YES
Constant	-0.0800	-1.295	-1.304	-2.815
	[0.142]	[0.933]	[0.964]	[1.734]
Antisocial punisher				
Condition	0.314	0.315	0.314	0.345
	[0.350]	[0.350]	[0.353]	[0.356]
Demographics	NO	YES	YES	YES
Preferences	NO	NO	YES	YES
Questionnaires	NO	NO	NO	YES
Constant	-1.872***	-2.425	-2.413	1.136
	[0.269]	[1.516]	[1.534]	[2.789]
Spiteful punisher				
Condition	1.048*	1.047*	1.097*	1.085*
	[0.428]	[0.428]	[0.437]	[0.438]
Demographics	NO	YES	YES	YES
Preferences	NO	NO	YES	YES
Questionnaires	NO	NO	NO	YES
Constant	-2.565***	-1.525	-1.460	-0.0677
	[0.367]	[1.791]	[1.865]	[3.183]
Observations	447	447	443	443

* $p < 0.05$, ** $p < 0.01$, *** $p < 0.001$

Standard errors in brackets

The table displays multinomial logit estimates for each individual modality of punishment (prosocial punishment, second-order free-riding, antisocial punishment, and spiteful punishment), as compared with the reference category of second-order free-riding (i.e., not taking a punishment action). Individual probit estimates for each modality of punishment separately obtained qualitative equivalent results (pooled data). The preferences group variable includes Falk's et al. (2018) Global Preferences survey proxies for altruism, positive reciprocity, negative reciprocity, trust, patience, and risk aversion. Finally, the questionnaire group variable includes the Buss-Perry Aggression Questionnaire, the Interpersonal Reactivity Index, and Barratt's Impulsiveness Scale.

References

1. J Dang, et al., A multilab replication of the ego depletion effect. *Soc. Psychol. Pers. Sci.* **12**, 14–24 (2021).
2. G Bernardi, et al., Neural and behavioral correlates of extended training during sleep deprivation in humans: evidence for local, task-specific effects. *J. neuroscience* **35**, 4487–4500 (2015).
3. A Quercia, F Zappasodi, G Committeri, M Ferrara, Local use-dependent sleep in wakefulness links performance errors to learning. *Front. Hum. Neurosci.* **12**, 122 (2018).
4. G Avvenuti, et al., Emotion regulation failures are preceded by local increases in sleep-like activity. *J. Cogn. Neurosci.* **33**, 2342–2356 (2021).
5. T Andrillon, A Burns, T Mackay, J Windt, N Tsuchiya, Predicting lapses of attention with sleep-like slow waves. *Nat. Commun.* **12**, 3657 (2021).
6. DJ Buysse, CF Reynolds III, TH Monk, SR Berman, DJ Kupfer, The pittsburgh sleep quality index: a new instrument for psychiatric practice and research. *Psychiatry research* **28**, 193–213 (1989).
7. JA Horne, O Östberg, A self-assessment questionnaire to determine morningness-eveningness in human circadian rhythms. *Int. J. Chronobiol.* **4**, 97–110 (1976).
8. A Falk, et al., Global evidence on economic preferences. *The Q. J. Econ.* **133**, 1645–1692 (2018).
9. JH Patton, MS Stanford, ES Barratt, Factor structure of the barratt impulsiveness scale. *J. Clin. Psychol.* **51**, 768–774 (1995).
10. AH Buss, M Perry, The aggression questionnaire. *J. Pers. Soc. Psychol.* **63**, 452–459 (1992).
11. MH Davis, A multidimensional approach to individual differences in empathy. *JSAS Catalog Sel. Documents Psychol.* **10**, 85 (1980).
12. D Watson, LA Clark, A Tellegen, Development and validation of brief measures of positive and negative affect: the panas scales. *J. Pers. Soc. Psychol.* **54**, 1063 (1988).
13. H Garavan, TJ Ross, EA Stein, Right hemispheric dominance of inhibitory control: an event-related functional mri study. *Proc. Proc. Natl. Acad. Sci.* **96**, 8310–8306 (1999).
14. H Garavan, TJ Ross, K Murphy, RA Roche, EA Stein, Dissociable executive functions in the dynamic control of behavior: inhibition, error detection, and correction. *NeuroImage* **17**, 1820–1829 (2002).
15. RA Roche, H Garavan, JJ Foxe, SM O'Mara, Individual differences discriminate event-related potentials but not performance during response inhibition. *Exp. brain research* **160**, 60–70 (2005).
16. YL Chuah, V Venkatraman, DF Dinges, MW Chee, The neural basis of interindividual variability in inhibitory efficiency after sleep deprivation. *J. Neurosci.* **26**, 7156–7162 (2006).
17. RF Baumeister, E Bratslavsky, M Muraven, DM Tice, Ego depletion: Is the active self a limited resource? *J. Pers. Soc. Psychol.* **74**, 1252–1265 (1998).
18. JR Stroop, Studies of interference in serial verbal reactions. *J. experimental psychology* **18**, 643 (1935).
19. JW Peirce, et al., Psychopy2: experiments in behavior made easy. *Behav. Res. Methods* (2019).
20. JW Peirce, RJ Hirst, MR MacAskill, Building experiments in psychopy. 2nd edn. (Sage, London), (2022).
21. J Dang, An updated meta-analysis of the ego depletion effect. *Psychol. research* **82**, 645–651 (2018).
22. DL Chen, M Schonger, C Wickens, otree—an open-source platform for laboratory, online, and field experiments. *J. Behav. Exp. Finance* **9**, 88–97 (2016).
23. J Brandts, G Charness, The strategy versus the direct-response method: A first survey of experimental comparisons. *Exp. Econ.* **14**, 375–398 (2011).
24. A Delorme, S Makeig, Eeglab: an open source toolbox for analysis of single-trial eeg dynamics including independent component analysis. *J. Neurosci. Methods* **134**, 9–21 (2004).
25. F Tadel, S Baillet, JC Mosher, D Pantazis, RM Leahy, Brainstorm: a user-friendly application for meg/eeg analysis. *Comput. intelligence neuroscience* pp. 1–12 (2011).
26. A Gramfort, T Papadopoulo, E Olivi, M Clerc, Openmeeg for m/eeg forward modeling: a comparison study in *Human Brain Mapping.* (7 June 2010).
27. TE Nichols, AP Holmes, Nonparametric permutation tests for functional neuroimaging: a primer with examples. *Hum. Brain Mapp.* **15**, 1–25 (2002).
28. CS Hung, et al., Local experience-dependent changes in the wake eeg after prolonged wakefulness. *Sleep* **36**, 59–72 (2013).
